# Supplementary material for: Selective Dehydration of Pentoses and Hexoses of Ulva rigida to Platform Chemicals Using Nb2O5 and ZrO2 Supported on Mesoporous Silicas as Heterogeneous Catalysts
Source: Int J Mol Sci. 2025 Oct 15;26(20):10054. doi: 10.3390/ijms262010054 (PMC12564892; doi:10.3390/ijms262010054)
Supplement: Supplementary file 1 [file ijms-26-10054-s001.zip › ijms-3875934-supplementary.pdf]

International Journal of *Molecular Sciences*

MDPI

Synthesis of the catalysts

1.1. Synthesis of SBA support

This synthesis is based on a published method [1]. Briefly, Pluronic-123 was dissolved in HCl 1.7 M until a homogeneous solution is obtained. Then, 17.5 mL of tetraethylortosilicate (TEOS) were added dropwise and was aged for 72 h. The white solid obtained was filtered, dried at 60 °C in a stove and calcined at 550 °C for 6h at 1 °C/min.

1.1. Synthesis of HMS support

The synthesis of HMS support followed a previous method developed by Cecilia et al. [2]. Firstly, 3.1 g of dodecylamine were dissolved into ethanol and 21.5 mL of water. Then, 14.8 mL of tetraethylortosilicate (TEOS) were added dropwise and the mixture was left to age for 24 h. The white solid obtained was filtered, dried at 60 °C and calcined at 550 °C for 6 h at 1 °C/min.

1.1. Impregnation of the catalysts

After the supports were synthesized, they were impregnated with the precursors of the metal oxides. For the impregnation of Nb_2O_5, the selected precursor was $Nb(HC_2O_4)_5$. 1.35 g of this precursor were dissolved in 0.1 M of oxalic acid and added into 2 g of support. When the support was SBA, the volume used was 5 mL while when the support was HMS, the volume was 6 mL, as those are their wet impregnation volumes. For the impregnation of ZrO_2, the precursor used was $ZrOCl_2$. 0.66 g of this precursor were dissolved in water and added into 2 g of support. For the catalysts that were impregnated with both species, they were firstly impregnated with one of the precursors and then with the second precursor. The quantities of precursors added were halved for these catalysts.

When the solids were correctly impregnated, they were dried overnight at 60 °C and calcined at 550 °C for 6 h at 1°C/min.

Identification and pretreatment of Ulva rigida

Molecular identification of the macroalga was performed by means of amplification and sequencing of the rbcL partial gene using primer pairs SHF1 and SHR4 [3]. The detailed identification of species-level and the phylogenetic analysis is described in Mascato et al. [4]. After the identification was performed, the alga was dried at 60 °C during 3 days in a stove and milled until desired particle size (0.2 mm).

Preparation of the Ulva rigida hydrolysate

For the preparation of the hydrolysate or liquor, an autohydrolysis in water was performed. 30 mg of macroalga were weighted and introduced into a 15 mL glass-lined reactor (Ace pressure®, Sigma Aldrich) along with 3 mL of water, so that the ratio of alga to solvent would be 1/100. The reactor was kept for 3 h at a temperature of 180 °C. After the reaction concluded, the solid rests were separated from the liquid phase. The liquid phase was then analyzed by means of HPLC. The results are presented in **Table S1**.

Table S1. Products present in the hydrolysate of *Ulva rigida* after autohydrolysis at 180 °C for 3 h.

Products	Cellobiose	Xylose	Rhamnose	Formic acid	Acetic acid	HMF	Furfural
mg/g$_{Ulva\ rigida}$	4.73	9.45	7.55	0.40	1.26	17.22	12.01

Total acid hydrolysis of the Ulva rigida hydrolysate

The total acid hydrolysis was used to calculate the potential hexoses and pentoses in the *Ulva rigida* liquor. The liquid phase recovered from the previous step underwent an acid treatment with sulphuric acid 4 vol% at 120 ºC during 3h, as proposed in El Harchi et al. [5]. and was subsequently measured through HPLC for the determination of the concentration of the monosaccharides. The results can be observed in **Table S2**.

Table S2. Products present in the hydrolysate of *Ulva rigida* after total hydrolysis at 120 °C for 3 h in the presence of 4 vol% H_2SO_4.

Products	Cellobiose	Glucose	Xylose	Rhamnose	Formic acid	Acetic acid	HMF	Furfural
mg/g*Ulva rigida*	22.45	71.87	63.27	110.37	2.24	4.87	2.67	0.28

X-ray photoelectron spectroscopy

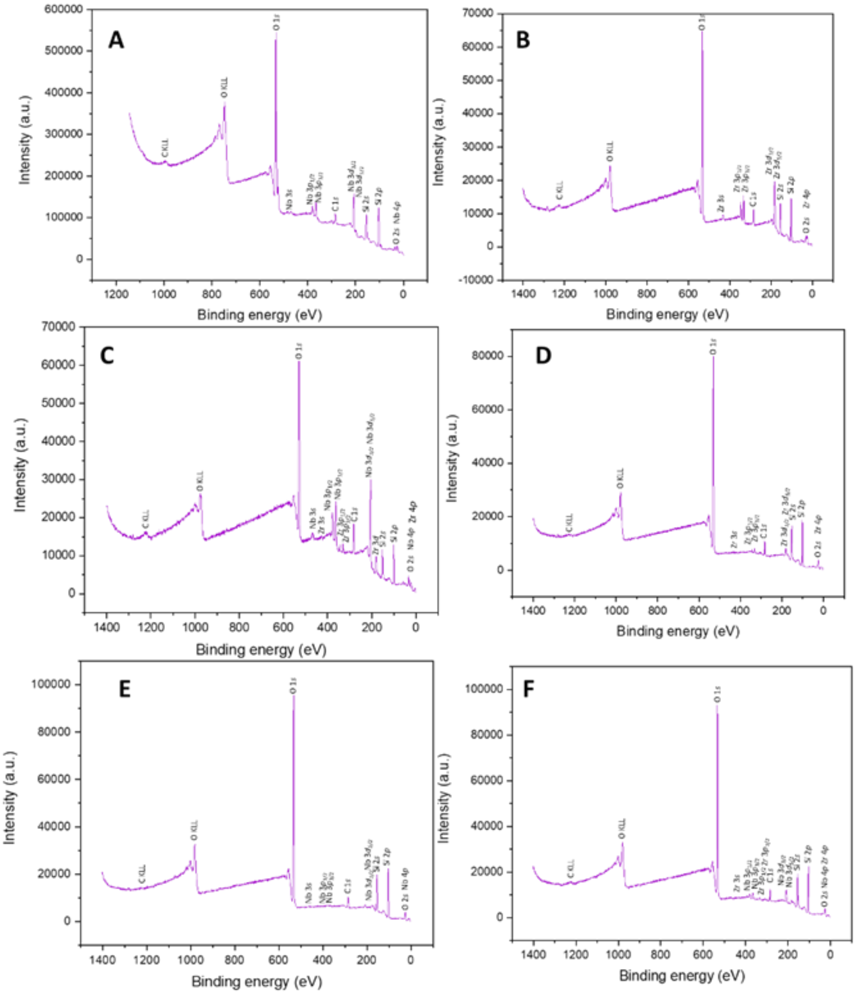

Figure S1. XPS survey spectra of **(A)** SBA-Nb, **(B)** SBA-Zr, **(C)** SBA-NbZr, **(D)** HMS-Zr.

Table S3. Binding energies of Nb and Zr core levels of the synthesized catalysts.

Catalyst	Nb 3*d*$_{3/2}$	Nb 3*d*$_{5/2}$	Zr 3*d*$_{3/2}$	Zr 3*d*$_{5/2}$
HMS-NbZr	210.2 (40.12%)	207.4 (59.88%)	185.3 (40.12%)	182.9 (59.88%)
HMS-Nb	210.7 (40.12%)	208.0 (59.88%)	--	--
HMS-Zr	--	--	185.4 (40.12%)	182.9 (59.88%)
SBA-NbZr	210.0 (40.12%)	207.3 (59.88%)	184.9 (40.12%)	182.4 (59.88%)

SBA-Nb	209.3 (40.12%)	206.5 (59.88%)	--	--
SBA-Zr	--	--	184.9 (40.12%)	182.5 (59.88%)

TEM and EDX images

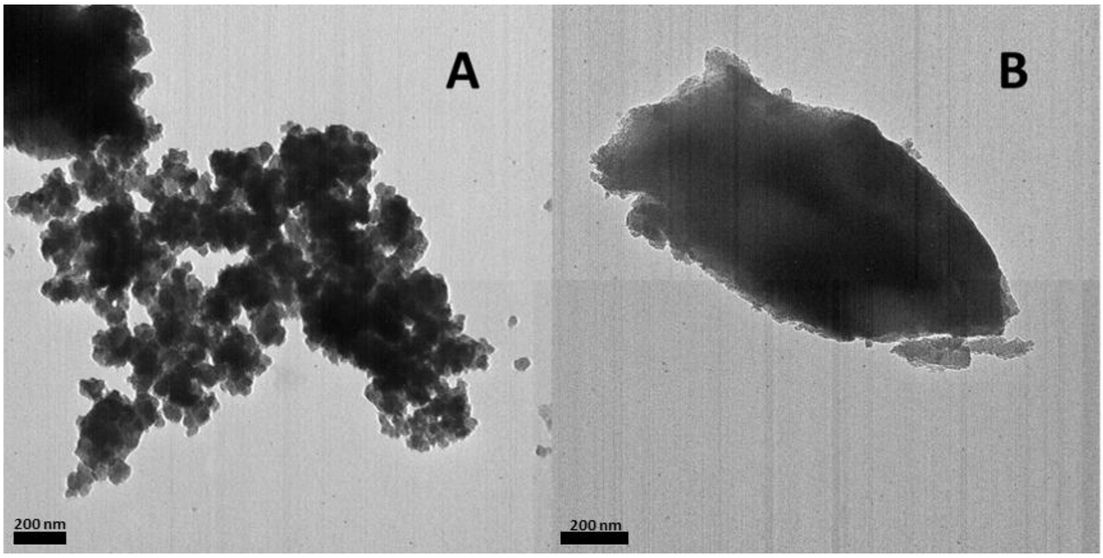

Figure S2. TEM micrographs of **(A)** HMS pristine support and **(B)** SBA pristine support.

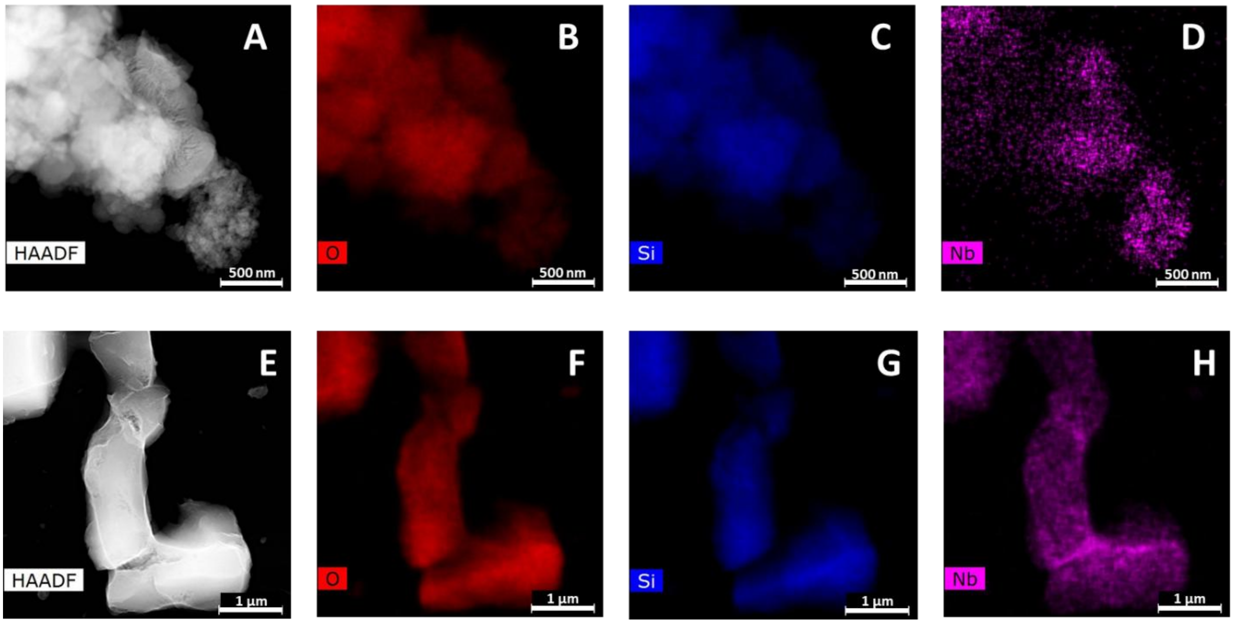

Figure S3. EDX mapping of **(A-D)** HMS-Nb and **(E-H)** SBA-Nb.

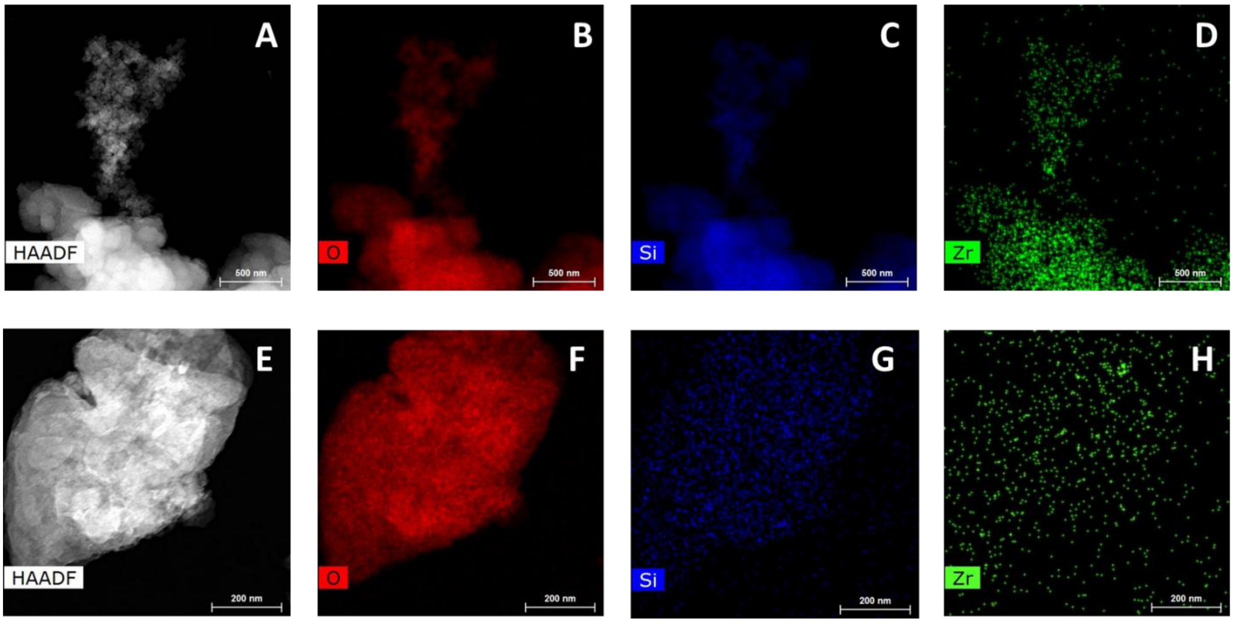

Figure S4. EDX mapping of **(A-D)** HMS-Zr and **(E-H)** SBA-Zr.

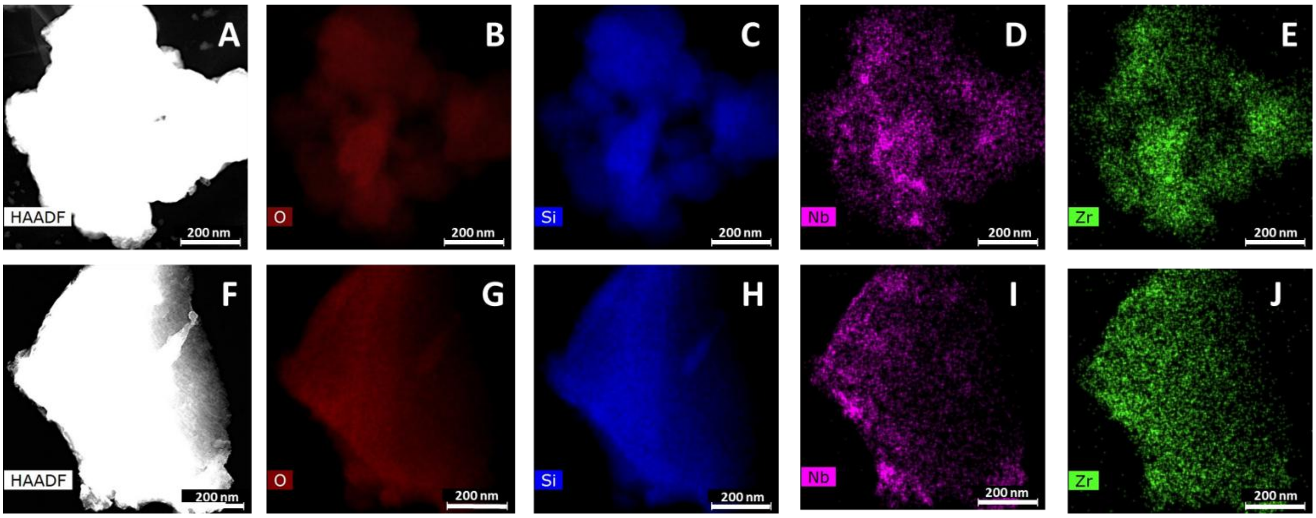

Figure S5. EDX mapping of **(A-E)** HMS-NbZr and **(F-J)** SBA-NbZr.

References

1. García-Sancho, C.; Saboya, R.; Cecilia, J.; Sales, A.; Luna, F.; Rodríguez-Castellón, E.; Cavalcante, C. Influence of pore size and loading for Nb 2 O 5 /SBA-15 catalysts on synthetic ester production from free fatty acids of castor oil. *Mol. Catal.* **2017**, *436*, 267–275, https://doi.org/10.1016/j.mcat.2017.04.036.
2. Cecilia, J.; Vilarrasa-García, E.; García-Sancho, C.; Saboya, R.; Azevedo, D.; Cavalcante, C.; Rodríguez-Castellón, E. Functionalization of hollow silica microspheres by impregnation or grafted of amine groups for the CO2 capture. *Int. J. Greenh. Gas Control.* **2016**, *52*, 344–356, https://doi.org/10.1016/j.ijggc.2016.07.018.
3. Heesch, S.; Broom, J.E.; Neill, K.F.; Farr, T.J.; Dalen, J.L.; Nelson, W.A. *Ulva*,*Umbraulva*and*Gemina*: genetic survey of New Zealand taxa reveals diversity and introduced species. *Eur. J. Phycol.* **2009**, *44*, 143–154, https://doi.org/10.1080/09670260802422477.
4. Massocato, T.F.; Robles-Carnero, V.; Moreira, B.R.; Castro-Varela, P.; Pinheiro-Silva, L.; Oliveira, W.d.S.; Vega, J.; Avilés, A.; Bonomi-Barufi, J.; Rörig, L.R.; et al. Growth, biofiltration and photosynthetic performance of Ulva spp. cultivated in fishpond effluents: An outdoor study. *Front. Mar. Sci.* **2022**, *9*, https://doi.org/10.3389/fmars.2022.981468.
5. El Harchi, M.; Kachkach, F.F.; El Mtili, N. Optimization of thermal acid hydrolysis for bioethanol production from Ulva rigida with yeast Pachysolen tannophilus. *South Afr. J. Bot.* **2018**, *115*, 161–169, https://doi.org/10.1016/j.sajb.2018.01.021.

6. Duan, G.; Zhang, C.; Li, A.; Yang, X.; Lu, L.; Wang, X. Preparation and Characterization of Mesoporous Zirconia Made by Using a Poly (methyl methacrylate) Template. *Nanoscale Res. Lett.* **2008**, *3*, 118–122, https://doi.org/10.1007/s11671-008-9123-7.

Disclaimer/Publisher's Note: The statements, opinions and data contained in all publications are solely those of the individual author(s) and contributor(s) and not of MDPI and/or the editor(s). MDPI and/or the editor(s) disclaim responsibility for any injury to people or property resulting from any ideas, methods, instructions or products referred to in the content.
